# Complications of miliary tuberculosis: low mortality and predictive biomarkers from a UK cohort

**DOI:** 10.1186/s12879-017-2397-6

**Published:** 2017-04-20

**Authors:** Jonathan Underwood, Fiona Cresswell, Alex P Salam, Alex J Keeley, Charles Cleland, Laurence John, Robert N Davidson

**Affiliations:** 1 Division of Infectious Diseases, Imperial College London, London, UK; 2 Lawson Unit, Brighton and Sussex University Hospitals NHS Trust, Brighton, UK; 3 King’s College London, London, UK; 4 Department of Medicine, Western Sussex Hospitals Trust, Worthing, UK; 5 Royal Free Hospital, London, UK; 6 Department of Infectious Diseases, Northwick Park Hospital, Northwest London Teaching Hospitals NHS Trust, London, UK

**Keywords:** Tuberculosis, Miliary tuberculosis, Central nervous system infection, Mortality, Biomarkers

## Abstract

**Background:**

Untreated, miliary tuberculosis (TB) has a mortality approaching 100%. As it is uncommon there is little specific data to guide its management. We report detailed data from a UK cohort of patients with miliary tuberculosis and the associations and predictive ability of admission blood tests with clinical outcomes.

**Methods:**

Routinely collected demographic, clinical, blood, imaging, histopathological and microbiological data were assessed for all patients with miliary TB identified from the London TB register from 2008 to 2012 from Northwest London Hospitals NHS Trust. Multivariable logistic regression was used to assess factors independently associated with the need for critical care intervention. Receiver operator characteristics (ROC) were calculated to assess the discriminatory ability of admission blood tests to predict clinical outcomes.

**Results:**

Fifty-two patients were identified with miliary tuberculosis, of whom 29% had confirmed central nervous system (CNS) involvement. Magnetic resonance imaging (MRI) was more sensitive than computed tomography (CT) or lumbar puncture for detecting CNS disease. Severe complications were frequent, with 15% requiring critical care intervention with mechanical ventilation. This was independently associated with admission hyponatraemia and elevated alanine aminotransferase (ALT). Having an admission sodium ≥125 mmol/L and an ALT <180 IU/L had 82% sensitivity and 100% specificity for predicting a favourable outcome with an area under the ROC curve (AUC) of 0.91. Despite the frequency of severe complications, one-year mortality was low at 2%.

**Conclusions:**

Although severe complications of miliary tuberculosis were frequent, mortality was low with timely access to critical care intervention, anti-tuberculous therapy and possibly corticosteroid use. Clinical outcomes could accurately be predicted using routinely collected biochemistry data.

## Background

Tuberculosis is one of the leading causes of death from infectious diseases worldwide [1]. Miliary tuberculosis results from widespread haematogenous spread of the *Mycobacterium tuberculosis* bacilli (TB). Clinical presentations are protean and vary from vague constitutional upset in ambulatory patients to abrupt multi-organ failure [2]. Untreated, miliary TB has a mortality approaching 100% [3], reducing to 7.1-30% with treatment [2, 4–6].

Miliary TB is uncommon, comprising 1-2% of TB cases [2], thus specific data regarding its management, particularly the ability to identify patients at high risk of poor outcomes, are lacking [2]. Current treatment guidelines are largely extrapolated from randomised controlled trials of pulmonary TB rather than specific trials of patients with miliary TB. As such, insights provided by cohort studies are important in guiding best practice. However, most cohorts are small and from the developing world, with limited neuroimaging and microbiological data. We sought to characterise the clinical course, investigation findings, and outcomes of a cohort with miliary TB from an ethnically diverse population with one of the highest TB incidences in the UK [7].

## Methods

### Participants

Patients with miliary TB were identified from the London TB register (LTBR) from 2008 to 2012 at Northwest London Hospitals NHS Trust. These records were cross-checked against the hospital database of all in-patient admissions.

### Data collection

Diagnosis of miliary TB was confirmed by identification of the characteristic miliary pattern on chest radiograph or computed tomography (CT) along with one or more of the following features: clinical features compatible with TB and response to anti-tuberculous therapy (ATT); histopathological evidence of TB; microbiological evidence of TB (positive smear or culture). Demographic, clinical, biochemical, radiological, histopathological evidence of TB (caseating or non-caseating granulomas with or without acid-alcohol fast bacilli on Ziehl-Neelson staining), and microbiological data were obtained from the LTBR and the hospital electronic patient records.

### Statistical analysis and biomarker selection

Univariate analysis of continuous data was performed using t-tests and the Mann-Whitney U tests as appropriate. For subsequent biomarker analysis, the primary outcome of interest, defined as an ‘unfavourable outcome’, was the need for critical care intervention with mechanical intervention. Whereas, a ‘favourable outcome’ was defined as not requiring critical care intervention with mechanical ventilation.

Multivariable logistic regression (*p* < 0.1 for entry from univariable logistic regression) was used to assess factors associated with the need for critical care intervention. Biomarkers that were independently associated with the need for critical care intervention (i.e. an ‘unfavourable outcome’) in the multivariable model were then assessed for their predictive performance by determining their receiver operator characteristics (ROC). Biomarker classification thresholds were determined by optimising the balance of sensitivity and specificity. Biomarker classification performance was assessed and thresholds determined using the area under the ROC curve (AUC). This is equivalent to the balanced accuracy of the classifier (i.e. accuracy taking account of differences in prevalence of the outcome between groups) for both ‘favourable’ (i.e. not requiring critical care intervention) and ‘unfavourable’ outcomes. Additionally, sensitivity, specificity, positive and negative predictive values were calculated. Confidence intervals were determined using bootstrapping with 1000 replicates.

Biomarkers were combined using an exhaustive grid across the range of all possible values and combinations to determine the optimum threshold for each biomarker in combination so that the AUC was maximised.

### Biomarker validation

Given the relatively small size of the dataset, splitting the data into separate training and testing datasets would be subject to bias. Therefore, to validate the predictive rule in this dataset, a leave-one-out-cross-validation (LOOCV) scheme was used. This entailed leaving one subject out of the dataset, then performing an exhaustive grid search, as before, to determine the optimal thresholds for the biomarker(s) of interest. This threshold(s) was then applied to the case that was left out. These steps were then repeated and summary statistics across the range of folds were calculated. Accuracy was determined by the percentage of correct responses across all the cross-validation folds.

## Results

### Baseline characteristics

Table 1 summarises the demographic data, investigations and clinical outcomes of the cohort of 52 patients with miliary TB. Thirty-six (69%) had typical miliary changes on chest radiographs with the remainder having miliary appearances confirmed on chest CT. The majority of patients (90%) were not born in the UK, had no significant comorbidity, and were nearly all (94%) vitamin D deficient. Half (14/24) had tuberculin skin test anergy and 6% were HIV co-infected. All but one patient (98%) were admitted to hospital with a median length of stay of 11 days (range 1 – 233 days).

**Table 1:** Baseline characteristics, co-morbidities, admission blood tests, neurological tests and outcomes of a UK cohort of patients with miliary tuberculosis

Variable	n (%) or median (range)
**Age** (years)	41 (10-87)
**Sex**	
Male	31 (60%)
**Ethnic Origin**	
Asian	32 (62%)
Black-African	11 (21%)
White	5 (10%)
Other	4 (8%)
**Previous diagnosis of TB**	2 (4%)
**Co-morbidities**	
None	33 (63%)
Diabetes	6 (12%)
CKD	4 (8%)
Anti-TNF therapy	3 (6%)
Alcoholism	3 (6%)
Heart failure	2 (4%)
HIV positive	3/49 (6%)
Hepatitis B sAg positive	3/49 (6%)
Hepatitis C positive	1/47 (2%)
Vitamin D deficient	44/57 (94%)
**Admission blood tests**	
Sodium (mmol/L)	132 (106-141)
Creatinine (mmol/L)	68 (44-559)
Alanine aminotransferase (IU/L)	46 (5-453)
Alkaline phosphatase (iU/L)	121 (39-866)
Bilirubin (μmol/L)	12 (4-39)
Erythrocyte sedimentation rate (mm/h)	33 (1-127)
C-reactive protein (mg/L)	56 (3-433)
White cell count (×10^9^/L)	7.1 (1.1-17.0)
Neutrophil count (×10^9^/L)	5.3 (0.9-15.7)
Platelet count (×10^9^/L)	238 (53-574)
Ferritin (μg/L)	522 (10-19,070)
Vitamin D (nmol/L)	16 (2-117)
**Assessment for CNS involvement**	
Number of patients assessed for CNS infection	29 (56%)
- CT head consistent with CNS infection	6/19 (32%)
- MRI brain consistent with CNS infection	10/11 (91%)
- Lumbar puncture consistent with CNS infection	4/17 (24%)
Total number with confirmed CNS infection	15 (29%)
**Outcomes and adverse events**	
Death (by one-year)	1 (2%)
Mechanical ventilation	8 (15%)
Drug-induced hepatitis	7 (13%)
Seizure	4 (8%)
Pneumothorax	3 (6%)
Haemophagocytic lymphohistiocytosis	2 (4%)
Pericardial effusion	2 (4%)

Data are presented as n (%), except for age and blood tests results which are presented as median (range)

*Abbreviations*: *TB* Tuberculosis, *CKD* chronic kidney disease, *TNF* tumour necrosis factor, *HIV* human immunodeficiency virus, *CNS* central nervous system, *CT* computer tomography; MRI: magnetic resonance imaging, *sAG* surface antigen

### Neuroimaging

Nineteen patients (37%) had a CT brain scan performed on their initial admission. Of these, 6 (31%) had ring-enhancing lesions typical of tuberculomas. Eleven patients (21%) underwent magnetic resonance imaging (MRI) of their brain, of whom 10 (91%) demonstrated tuberculomas. Notably, 4 patients with normal/nonspecific admission CT had subsequent MRI because due to clinical concern of neurological disease, all of whom demonstrated tuberculomas. The overall prevalence of tuberculomas seen on MRI or CT was 12 of 23 (52%), but we may have missed lesions in the 29 patients who did not undergo neuroimaging, and in patients in whom tuberculomas developed subsequently.

### Microbiology

Forty patients (77%) had positive cultures for *M. tuberculosis* (MTB) complex. Induced sputa lead to a positive culture in thirty (58%) patients. Only ten sputum samples (19%) revealed acid-alcohol fast bacilli (AFB) on microscopy. Seventeen patients (33%) had cerebrospinal fluid (CSF) examination. Of these, four (24%) had findings compatible with TB meningitis, all of whom subsequently cultured MTB (two of four [50%] had tuberculomas). Aside from sputa and CSF, MTB was cultured from joint (*n* = 2),  pericardial (*n* = 2) and pleural (*n* = 1) fluids (*n* = 1), transbronchial biopsy (*n* = 1) and bone marrow (*n* = 1). Four (10%) were resistant to isoniazid, three (6%) to pyrazinamide, three (6%) to streptomycin, one to rifampicin and isoniazid (i.e. multidrug resistant – MDR) and none to ethambutol.

### Treatment

All patients were commenced on quadruple ATT with most receiving rifampicin, isoniazid, pyrazinamide and ethambutol. Median (range) duration of ATT was 12 (1.5 – 28) months. Ten (19%) patients stopped pyrazinamide due to drug-induced liver injury (15%) or intolerance (4%). 29 patients (56%) received corticosteroids during their ATT for a variety of indications including central nervous system (CNS) involvement, systemic inflammatory response syndrome and paradoxical reactions.

### Complications

Follow-up to one-year was complete for 49 patients (94%). One 87-year-old patient with multiple co-morbidities, including chronic renal failure, died during treatment (after 1.5 months’ therapy). One patient died subsequently from constrictive pericarditis secondary to pericardial involvement, two years after successfully completing 12 months of ATT.

Eight patients (15%) required critical care intervention with mechanical ventilation, seven with respiratory failure and one with neurologic deterioration. Other serious adverse events are shown in Table 1. Corticosteroid use was more frequent (88% vs. 50%, *p* = 0.11) in patients that required mechanical ventilation. In a univariate model, requirement for mechanical ventilation was associated with thrombocytopenia (median platelet count in those needing: 148 × 10^9^/L vs. not needing mechanical ventilation: 266 × 10^9^/L, *p* = 0.04), hyponatraemia (125 vs. 133 mmol/L, *p* = 0.03), hyperbilirubinaemia (17 vs. 11 μmol/L, *p* < 0.01), and increased alanine aminotransferase (ALT: 196 vs. 35 IU/L, *p* < 0.001). There were borderline associations with increased erythrocyte sedimentation rate (37 vs. 18 mm/h, *p* = 0.08) and c-reactive protein (97 vs 48 mg/L, *p* = 0.06) among those requiring mechanical ventilation. There was no significant association between requirement for mechanical ventilation and age (*p* = 0.72), admission vitamin D (*p* = 0.39), total white cell count (*p* = 0.14), neutrophil count (*p* = 0.45), creatinine (*p* = 0.34), alkaline phosphatase (*p* = 0.53) nor ferritin (*p* = 0.54 but data were only available for 23/52). Additionally, neither comorbidity nor ethnicity were associated with the need for critical care intervention (*p* > 0.8 for all). Whilst there was a trend for a difference in the distribution of ESR between those who did and did not require critical care intervention, in a univariable logistic regression model it was not predictive (odds ratio [95% confidence intervals]: 0.98 [0.94-1.01] per 100 mm/h; *p* = 0.19). Therefore, ESR was not included in the multivariable model. In a multivariable logistic regression model, only sodium (OR [95% CIs] 0.19 [0.02-0.93] per 10 mmol/L reduction, *p* = 0.04) and an ALT (1.33 [1.11-1.78] per 10 IU/L increase, *p* < 0.0001) were independently associated with the need for mechanical ventilation (Table 2).

**Table 2:** Univariable and multivariable logistic regression model results for the outcome of requiring critical care intervention

Parameter	Univariable		Multivariable	
	Odds ratio (95% CI)	*p*-value	Odds ratio (95% CI)	*p*-value
ALT (per 10 IU/L)	1.29 (1.13-1.56)	<0.001	1.33 (1.11-1.78)	<0.001
Bilirubin (per 10 μmol/L)	2.99 (1.29-7.84)	0.01	0.69 (0.07-3.86)	0.68
CRP (per 10 mg/L)	1.14 (1.02-1.35)	0.02	1.13 (0.93-1.61)	0.27
Platelet count (per 100 cells/mm3)	0.39 (0.14-0.87)	0.02	0.75 (0.13-3.60)	0.71
Sodium (per 10 mmol/L)	0.40 (0.14-1.03)	0.06	0.19 (0.02-0.93)	0.04

*Abbreviations*: *ALT* alanine aminotransferase, *CI* confidence intervals; *CRP* c-reactive protein

**Fig. 1:**
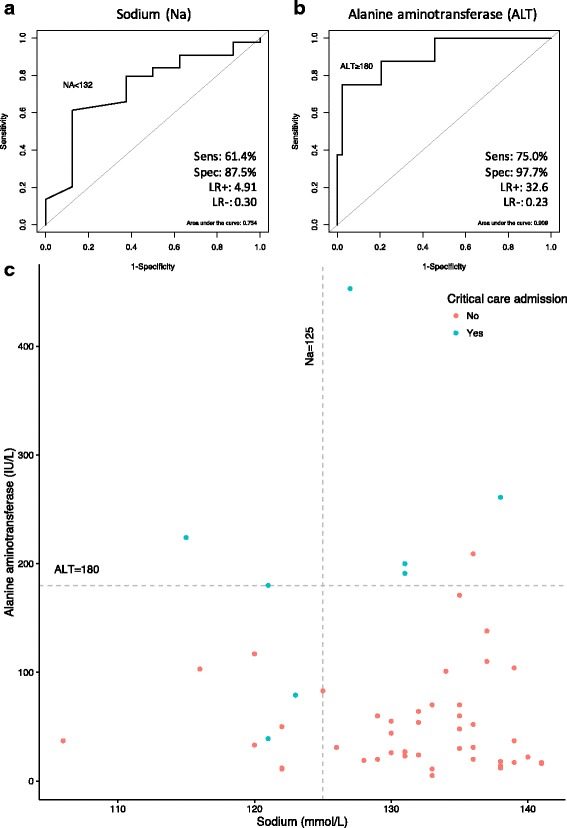
Predictive characteristics of admission sodium and alanine aminotransferase for adverse clinical outcomes. Top: Receiver operator characteristics of admission sodium (**a**) and alanine aminotransferase (**b**) for predicting the need for critical care intervention with mechanical intervention with displayed statistics. Bottom: **c** graphically depicts patients by need for critical care intervention with cut-offs for admission sodium and alanine aminotransferase that identify a favourable outcome with 100% specificity. Abbreviations: ALT: alanine aminotransferase; Na: sodium; Sens: sensitivity; Spec: specificity; LR+: positive likelihood ratio; LR-: negative likelihood ratio

Admission ALT and sodium were both independently associated with the need for critical care intervention with mechanical ventilation and therefore underwent further ROC analysis to determine predictive thresholds. For the independent prediction of an unfavourable outcome, the optimal threshold for admission ALT was ≥180 IU/L and for admission sodium was <132 mmol/L (Fig. 1 and Table 3). The addition of sodium to ALT did not alter classification performance, suggesting that using ALT ≥180 IU/L is sufficient to predict an unfavourable outcome with a positive likelihood ratio of 32.6. Conversely, for the prediction of a favourable outcome, a different threshold for sodium was evident and classification performance was improved by combining ALT <180 IU/L (individual AUC: 0.864) and sodium ≥125 mmol/L (individual AUC: 0.670) thresholds (combined AUC 0.909, Table 3). It should be noted that this is equivalent to using an ALT threshold ≥180 IU/L *or* sodium <125 mmol/L as criteria from predicting an unfavourable outcome. The relationship between admission sodium and ALT with outcomes is displayed graphically in Fig. 1.

**Table 3:** Classification performance of different biomarkers and their optimum threshold by outcome determined using bootstrapping

Predictor	Outcome	Sensitivity (%)	Specificity (%)	PPV (%)	NPV (%)	AUC
ALT ≥180 IU/L	Unfavourable	75.0 (41.7-100)	97.3 (92.5-100)	85.7 (50.0-100)	95.6 (88.6-100)	0.864 (0.692-1.00)
Sodium <132 mmol/L	Unfavourable	87.5 (57.1-100)	61.4 (46.5-76.1)	29.2 (11.1-48.3)	96.4 (88.9-100)	0.744 (0.583-0.869)
ALT <180 IU/L & sodium ≥125 mmol/L	Favourable	81.8 (69.8-92.5)	100 (100-100)	100 (100-100)	50.0 (25.0-76.9)	0.909 (0.849-0.963)

An unfavourable outcome was defined as the need for critical care intervention with mechanical ventilation, whereas a favourable outcome was defined as not requiring critical care intervention with mechanical ventilation. 95% confidence intervals determined by bootstrapping (1000 replicates) are in parentheses for each parameter. *Abbreviations*: *ALT* alanine aminotransferase, *AUC* area under the receiver operator curve (equivalent to the balanced accuracy), *PPV* positive predictive value, *NPV* negative predictive value

Given the size of the dataset, biomarker prediction validity was assessed using LOOCV. For the prediction of an unfavourable outcome the median (range) ALT threshold was: ≥180 (180-191) IU/L with diagnostic accuracy of 92.3% across the cross-validation folds. For the prediction of a favourable outcome, the median (range) ALT threshold was <191 (180-200) IU/L; and for sodium was: ≥125 (106-126) mmol/L yielding a diagnostic accuracy of 76.9% across the cross-validation folds.

## Discussion

We report the lowest one-year mortality from miliary TB (2%), despite significant rates of severe complications. Additionally, we demonstrate how routinely collected biochemical measures can be used to accurately predict clinical outcomes.

The mortality rate in our cohort compares favourably to previously reported rates of 10-30% from the developing world and 7.1%-21% from the developed world [2, 4–6]. Critical care intervention and mechanical ventilation, which was required in 15% of our cohort, are likely to be responsible for the low mortality we report as these patients would almost certainly have died if these facilities were not available. Additionally, extensive experience in the management of complicated tuberculosis at a high-volume centre are likely to have contributed to our low mortality. Due to the retrospective nature of data collection it was not possible to assess the impact of corticosteroid usage on outcome, and whilst corticosteroid usage was higher in patients requiring mechanical ventilation this may reflect increased rates of CNS involvement and systemic inflammatory response syndrome in patients requiring critical care support. The impact of corticosteroids on mortality in CNS TB infection has been demonstrated, particularly in non HIV-infected individuals [8]. Furthermore, meta-analysis of corticosteroid use in all forms of TB suggest a beneficial effect on mortality, however many of the 41 randomised controlled trials were identified as being at high risk of bias, and none were performed to assess corticosteroid usage specifically in miliary TB [9]. Given the multisystem nature of miliary TB with frequent and potentially under-recognised CNS involvement, further research is warranted to clarify the role of corticosteroids.

Hyponatraemia and an elevated ALT on admission were associated with an unfavourable clinical course. The causes of hyponatraemia in those with miliary TB are numerous, but are likely to include brain injury as well as adrenal and pituitary dysfunction. Whereas, an increased ALT identifies those with significant liver injury and in this cohort a threshold of ≥180 IU/L was strongly predictive of an unfavourable outcome. Both factors are likely surrogates of disease burden, hence their association with clinical outcomes. Our data suggests these routinely collected biochemical measures could be used to identify patients with severe disease at risk of deterioration. However, these thresholds need verifying in an independent dataset. Pending further study, they should be interpreted with caution given the relatively low number of patients in our study. Furthermore, the influence of co-morbidity, in particular HIV-infection and type 2 diabetes given their increasing prevalence in regions with the highest burden of tuberculosis, on the relationship between predictive biomarkers and outcomes needs to be determined.

UK National Institute for Health and Care Excellence (NICE) guidelines [10] currently recommend neuroimaging only for miliary TB patients with CNS signs or symptoms. Given that miliary TB is caused by the widespread haematogenous dissemination of *MTB*, it is unsurprising that CNS involvement was proven in 29% of our cohort. However, as not all our patients underwent systematic neuroimaging or CSF sampling, this represents the lower bound of the true prevalence of CNS disease and confirms historical autopsy data documenting high rates of CNS disease [11]. In keeping with other studies, MRI was more sensitive than CT in detecting tuberculomas [4]. CSF examination demonstrated TB meningitis in a minority, suggesting that meningitis may be a late manifestation of disease, however, normal CSF biochemistry and microscopy did not exclude CNS involvement. In addition, CNS tubercles may initially be below the size of detection by CT or MRI, and may paradoxically appear during treatment.

There are no randomised trials to provide guidance on duration of therapy for miliary TB. A 6-month course is currently recommended for miliary TB without CNS involvement in UK, USA, Europe and WHO international guidelines [10, 12–14]. However, as we suspect CNS involvement in miliary TB is often under-recognised, a 6-month course of ATT may risk later relapse and complications. Generally, patients in our cohort were treated for 12-months. Whether this contributed to our low mortality is unclear. Given the ubiquity of CNS involvement, we advocate performing contrast-enhanced neuroimaging (ideally MRI) in all patients and CSF examination in those with symptoms or signs of meningitis. Ideally, this should be at the time of diagnosis and possibly repeated 6-12 weeks later to detect tuberculomas which appear during treatment [15]. In settings where this is not possible, 9-12 months’ treatment to cover CNS disease may be prudent. Given the high *a priori* probability of CNS disease, we advocate consideration for longer empirical duration of therapy (9-12 months) if contrast-enhanced MRI and CSF examination cannot be undertaken, such as in the developing world.

The limitations of this case series include the retrospective and non-systematic nature of initial data collection and incomplete post-treatment follow-up. However, most mortality attributed to miliary TB occurs in the early stages of disease so it is unlikely that longer follow-up would significantly change our conclusions [16, 17]. Additionally, due to the low mortality in our cohort, where there was only one death, we lacked power to detect associations with this important outcome.

## Conclusions

CNS involvement in miliary TB is common and is likely to be under-recognised and under-treated. Although severe complications occur frequently, they can be predicted by admission hyponatraemia and increased ALT. A low one-year mortality is achievable in a high resource setting with critical care support and mechanical ventilation. Further research is required to establish the role of corticosteroids and prolonged ATT in the management of miliary TB.

## Abbreviations

ALT: Alanine aminotransferase
ATT: Anti-tuberculous therapy
CNS: Central nervous system
CSF: Cerebrospinal fluid
CT: Computer tomography
HIV: Human immunodeficiency virus
MRI: Magnetic resonance imaging
MTB: *Mycobacterium tuberculosis*
TB: Tuberculosis

## References

[1] Murray CJ, Lopez AD (1997). Global mortality, disability, and the contribution of risk factors: global burden of disease study. Lancet.

[2] Sharma SK, Mohan A, Sharma A, Mitra DK (2005). Miliary tuberculosis: new insights into an old disease. Lancet Infect Dis.

[3] Falk A (1965). U. S. veterans administration-armed forces cooperative study on the chemotherapy of tuberculosis. Tuberculous meningitis in adults, with special reference to survival, neurologic residuals, and work status. Am Rev Respir Dis.

[4] Venkatraman N, King T, Bell D, Woltmann G, Wiselka M, Abubakar I (2016). High levels of neurological involvement but low mortality in miliary tuberculosis: a 6-year case-series from the UK. Eur Respir J.

[5] Ormerod LP, Horsfield N (1995). Miliary tuberculosis in a high prevalence area of the U.K.: Blackburn 1978-1993. Respir Med.

[6] Touré NO, Cissé MF, Dia Kane Y, Diatta A, Bouker Bakioui B, Ndiaye EHM (2011). Miliary tuberculosis: a report of 49 cases. Rev Mal Respir.

[7] Tuberculosis in the UK, 2014 report. Public Health England (https://www.gov.uk/government/uploads/system/uploads/attachment_data/file/360335/TB_Annual_report__4_0_300914.pdf); 2014.

[8] Prasad K, Singh MB, Ryan H (2016). Corticosteroids for managing tuberculous meningitis. Cochrane Database Syst Rev.

[9] Critchley JA, Young F, Orton L, Garner P (2013). Corticosteroids for prevention of mortality in people with tuberculosis: a systematic review and meta-analysis. Lancet Infect Dis.

[10] Tuberculosis. National Institute for Health and Care Excellence; 2016. (https://www.nice.org.uk/guidance/ng33/).

[11] American College of Physicians. Tuberculoma of the brain. Ann Intern Med. 1934;7:1141–5.

[12] American Thoracic Society - AJRCCM. American Thoracic Society/Centers for Disease Control and Prevention/Infectious Diseases Society of America. Am J Respir Crit Care Med. 2003;167:603–62. 10.1164/rccm.167.4.603 12588714

[13] Migliori GB, Zellweger JP, Abubakar I, Ibraim E, Caminero JA, De Vries G (2012). European union standards for tuberculosis care. Eur Respir J.

[14] World Health Organization. Guidelines for treatment of tuberculosis. WHO. World Health Organization; 2010. (http://www.who.int/tb/publications/2010/9789241547833/en/).

[15] Afghani B, Lieberman JM (1994). Paradoxical enlargement or development of intracranial tuberculomas during therapy: case report and review. Clin Infect Dis.

[16] Lin C-H, Lin C-J, Kuo Y-W, Wang J-Y, Hsu C-L, Chen J-M (2014). Tuberculosis mortality: patient characteristics and causes. BMC Infect Dis BioMed Central.

[17] Gelb AF, Leffler C, Brewin A, Mascatello V, Lyons HA. Miliary Tuberculosis. American Review of Respiratory Disease. Am Lung Assoc. 1973;108(6):1327–33. 10.1164/arrd.1973.108.6.1327 4201630

